# Supplementary material for: Ancient Mitogenomes Reveal Stable Genetic Continuity of the Holocene Serows
Source: Genes (Basel). 2023 May 29;14(6):1187. doi: 10.3390/genes14061187 (PMC10297833; doi:10.3390/genes14061187)
Supplement: Supplementary file 1 [file genes-14-01187-s001.zip › genes-2405699-supplementary.pdf]

Supporting Information

Figure S1. Photos of two serow sub-fossil specimens. (a): CADG946, (b): CADG839.

Figure S2. Bayesian phylogenetic tree of mitochondrial genomes in the Bovidae family. The calibration points are indicated with an asterisk. Node values are divergence times and node bars are the 95% bounds of the highest posterior density (95% HPD).

Figure S3. DNA damage signal of two serow sub-fossil specimens (a) Mitochondrial read length distribution of two serow sub-fossil specimens. (b) Mitochondrial DNA damage plots for four two serow sub-fossil specimens. *X* axis represents position from 5’ (left) and 3’ (right) read ends. Red line corresponds to C to T substitutions and blue line corresponds to G to A substitutions.

Figure S4. The phylogenetic tree constructed using the Maximum-Likelihood (ML) and Neighbor-Joining (NJ) methods, with support values for the main nodes indicated below the nodes (ML/NJ).

Table S1. Information of two serow sub-fossil specimens.

Table S2. Number of reads that match to different reference genomes of serows.

Table S3. Quality information of the sequencing libraries.

Table S4. Information of data set used in this study.

Table S5. Mitochondrial genome information of serows used in this study. *** means “No information found”.

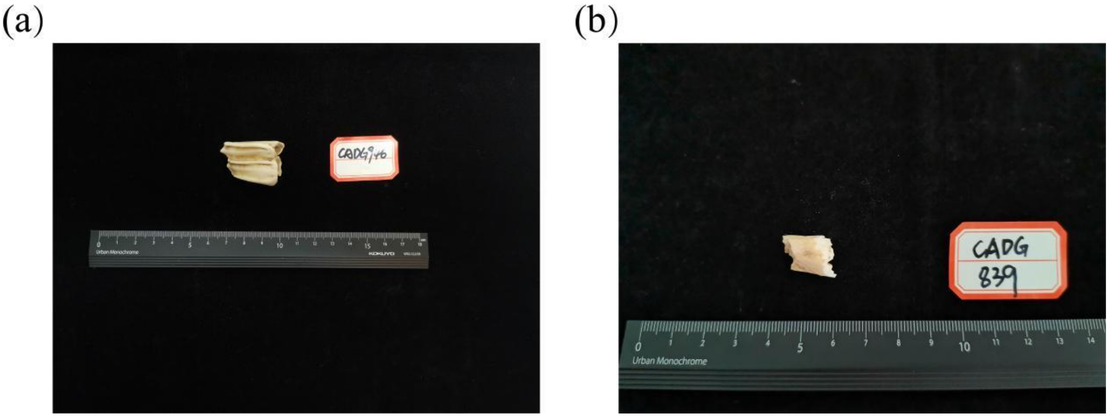

Figure S1. Photos of two serow sub-fossil specimens. (a): CADG946, (b): CADG839.

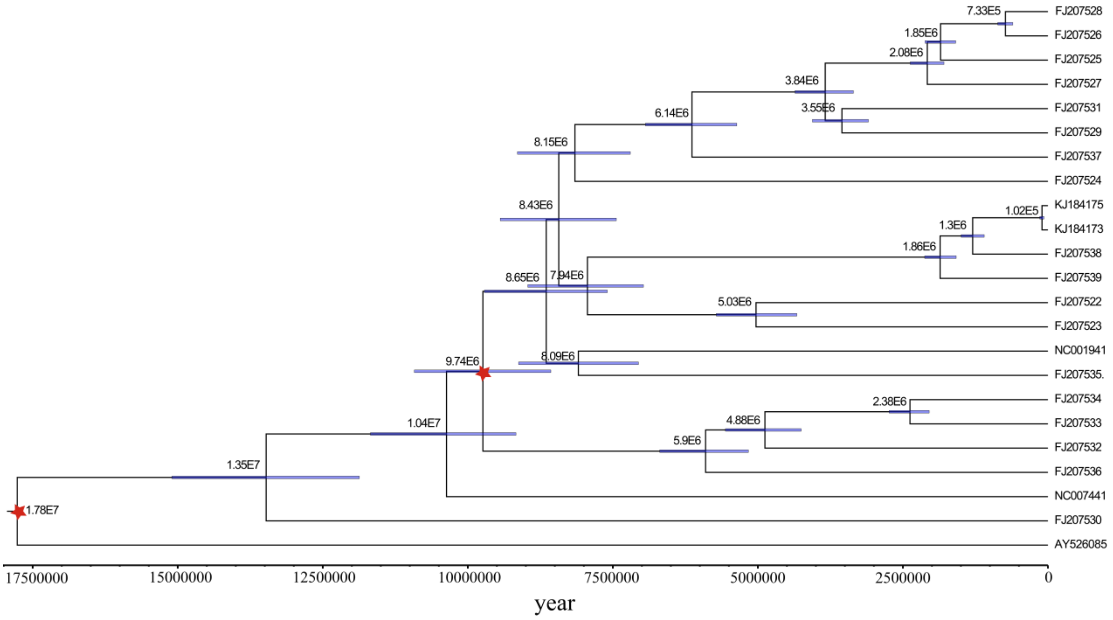

Figure S2. Bayesian phylogenetic tree of mitochondrial genomes in the Bovidae family. The calibration points are indicated with an asterisk. Node values are divergence times and node bars are the 95% bounds of the highest posterior density (95% HPD).

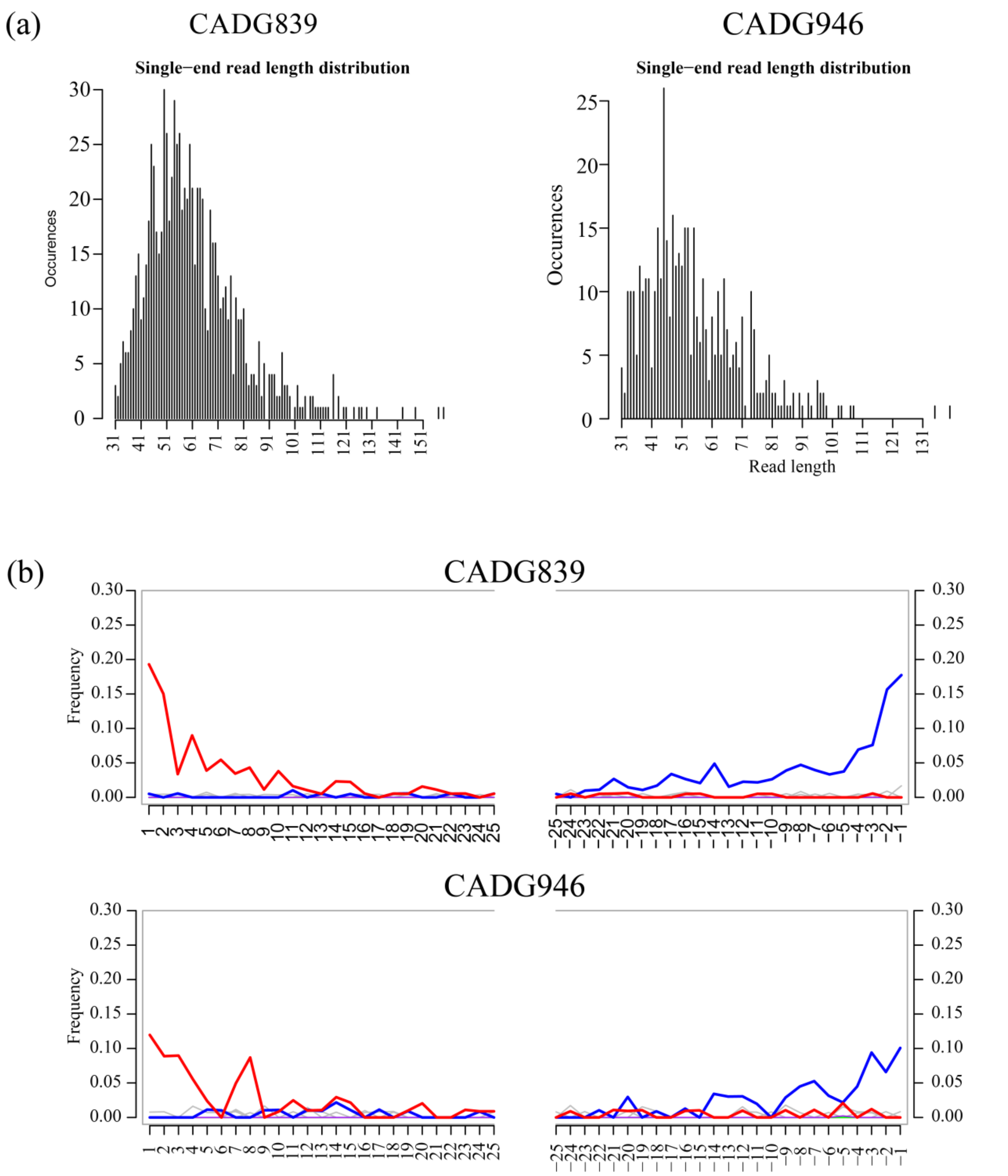

Figure S3. DNA damage signal of two serow sub-fossil specimens (a) Mitochondrial read length distribution of two serow sub-fossil specimens. (b) Mitochondrial DNA damage plots for four two serow sub-fossil specimens. *X* axis represents position from 5’ (left) and 3’ (right) read ends. Red line corresponds to C to T substitutions and blue line corresponds to G to A substitutions.

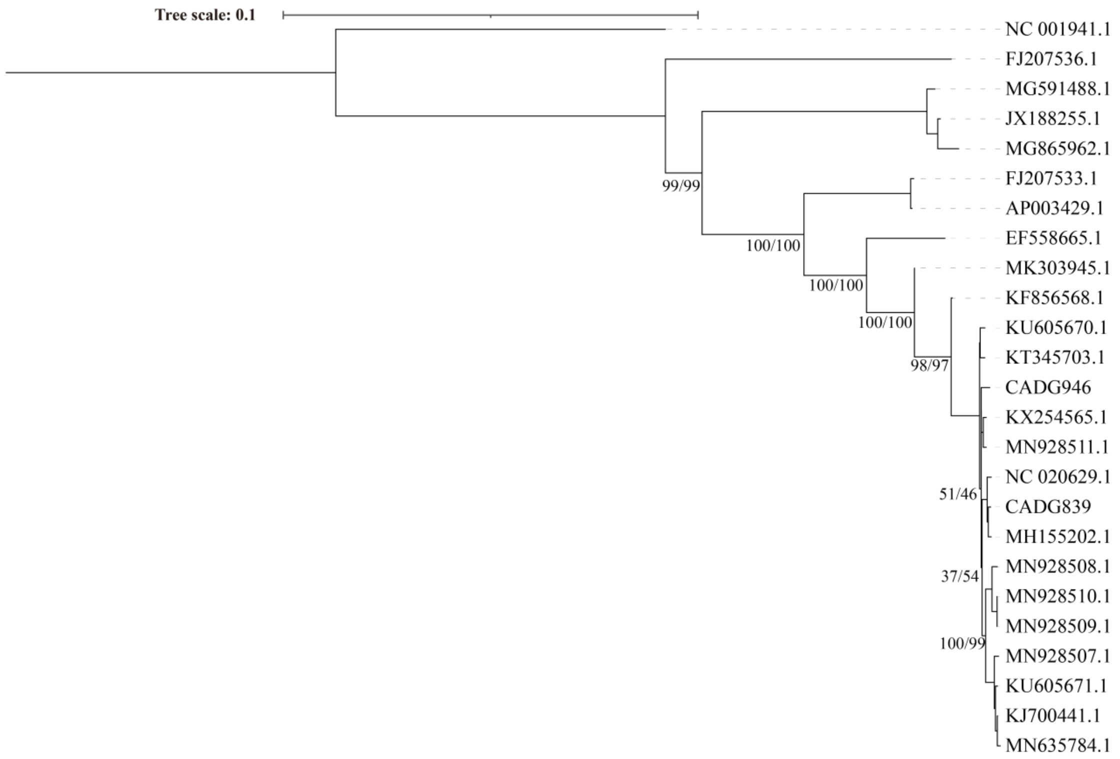

Figure S4. The phylogenetic tree constructed using the Maximum-Likelihood (ML) and Neighbor-Joining (NJ) methods, with support values for the main nodes indicated below the nodes (ML/NJ).

Table S1. Information of two serow sub-fossil specimens.

Sample No.	Skeletal element	Location	Radiocarbon age (14C, BP)	Calibrated radiocarbon age (BP)	Sequence length (bp)	Average read depth
CADG 839	tooth	Beijing city	8,860 ± 30	95.4% (9884-10160)	15577	8.13
CADG 946	tooth	Guizhou province	2,450 ± 30	95.4% (2361-2703)	15597	7.98

Table S2. Number of reads that match to different reference genomes of serows.

Library No.	Mapping to *C.sumatraensis*	Mapping to *C. rubidus*	Mapping to *C. crispus*	Mapping to *C. swinhoei*
839	136 (130)	101 (95)	71 (67)	86 (80)
839X1	43 (42)	37 (36)	22 (21)	31 (30)
839X2	125 (114)	97 (87)	69 (36)	87 (78)
839X3	750 (642)	600 (512)	429 (362)	536 (455)
D839X1	194 (163)	146 (119)	114 (98)	127 (103)
D839X3	1184 (1012)	933 (793)	685 (590)	835 (708)
946	15 (14)	14 (13)	12 (11)	14 (13)
946X1	265 (176)	221 (148)	164 (108)	190 (137)
946X2	131 (65)	101 (51)	85 (41)	77 (39)
946X3	170 (137)	136 (108)	105 (86)	136 (108)
946X4	2645 (1653)	2149 (1359)	1635 (1030)	1996 (1241)
946X5	464 (385)	387 (319)	263 (220)	327 (271)
D946X1	289 (176)	238 (145)	174 (108)	225 (137)
D946X2	71 (49)	57 (40)	41 (27)	47 (32)
D946X3	315 (279)	254 (223)	173 (156)	239 (211)

Table S3. Quality information of the sequencing libraries.

Library No.	Total reads	Mapped reads	Uniq mapped reads	Mapped bp	Duplication
839	6835615	136	130	7648	0.044
839X1	2940297	43	42	2515	0.023
839X2	10092338	125	114	7794	0.088
839X3	8153171	750	642	32997	0.144
D839X1	11501953	194	163	9629	0.16
D839X3	12836964	1184	1012	52758	0.145
946	6689223	15	14	861	0.067
946X1	15728523	265	176	9335	0.336
946X2	6699924	131	65	4096	0.504
946X3	7796900	170	137	5939	0.194
946X4	107429218	2645	1653	71016	0.375
946X5	7776947	464	385	23249	0.17
D946X1	14131200	289	176	9138	0.391
D946X2	3369630	71	49	2884	0.31
D946X3	13291798	315	279	12151	0.114

Table S4. Information of data set used in this study.

Data set for analysis	Number in Genbank
Bayesian phylogenetic MCC tree and the ML phylogenetic tree	CADG946, CADG839, KU605670, KT345703, KX254565, MH155202, KF856568, KJ700441, MN635784, KU605671, MN928511, MN928510, MN928509, MN928508, MN928507, MK303945, EF558665, FJ207533, AP003429, NC020629, NC001941, FJ207536, MG591488, MG865962, JX188255
Median-joining network	CADG946, CADG839, KU605670, KT345703, KX254565, MH155202, KF856568, KJ700441, MN635784, KU605671, MN928511, MN928510, MN928509, MN928508, MN928507, MK303945, EF558665, FJ207533, AP003429, NC020629
The Bayesian skyline plot	CADG946, CADG839, KU605670, KT345703, KX254565, MH155202, KJ700441, MN635784, KU605671, MN928511, MN928510, MN928509, MN928508, MN928507, NC020629, KF856568

Table S5. Mitochondrial genome information of serows used in this study. *** means "No information found".

Number	Location	reference	Reference Status
NC020629	Cambodia	Evolution of the mitochondrial genome in mammals living at high altitude: new insights from a study of the tribe Caprini (Bovidae, Antilopinae)	Published
AP003429	Japan	Mammal complete mitochondrial genome	Unpublished
FJ207533	Japan	Evolution of the mitochondrial genome in mammals living at high altitude: new insights from a study of the tribe Caprini (Bovidae, Antilopinae)	Published

EF558665	Taiwan, China	The complete sequence of Formosan serow mitochondrial genome	Unpublished
MK303945	Assam, India	Reclassification of the serows and gorals: the end of a neverending story?	Published
MN928507	Northern Myanmar	Comparative biogeography of serow species complex and gorals in northern Myanmar based on mitogenome	Unpublished
MN928508	Northern Myanmar	Comparative biogeography of serow species complex and gorals in northern Myanmar based on mitogenome	Unpublished
MN928509	Northern Myanmar	Comparative biogeography of serow species complex and gorals in northern Myanmar based on mitogenome	Unpublished
MN928510	Northern Myanmar	Comparative biogeography of serow species complex and gorals in northern Myanmar based on mitogenome	Unpublished
MN928511	Northern Myanmar	Comparative biogeography of serow species complex and gorals in northern Myanmar based on mitogenome	Unpublished
KU605671	South China	***	Direct Submission
MN635784	Guizhou, China	Characteristic of complete mitochondrial genome and phylogenetic relationship of a Chinese Serow in Xishui, China	Published
KJ700441	Guizhou, China	The complete mitochondrial genome of Capricornis sp., possible a new species of Serow from Guizhou, China	Published
KF856568	Sichuan, China	The complete mitochondrial genome sequence of the Chinese Serow, Capricornis	Published

		milneedwardsii (Cetartiodactyla: Caprinae)	
MH155202	Sichuan, China	The complete mitochondrial genome sequence and phylogenetic analysis of Chinese Serow (Capricornis Milneedwardsii)	Published
KX254565	***	***	Direct Submission
KT345703	Tibet, China	Complete mitochondrial genome of the Himalayan serow (Capricornis thar) and its phylogenetic status within the genus Capricornis	Published
KU605670	South China	***	Direct Submission
CADG839	Beijing, China	This study	
CADG946	Guizhou, China	This study	
